# Prevention of Dielectric Breakdown of Nanopore Membranes by Charge Neutralization

**DOI:** 10.1038/srep17819

**Published:** 2015-12-04

**Authors:** Kazuma Matsui, Itaru Yanagi, Yusuke Goto, Ken-ichi Takeda

**Affiliations:** 1 Hitachi, Ltd., Research & Development Group, Center for Technology Innovation - Healthcare, 1-280 Higashi-koigakubo, Kokubunji, Tokyo, 185-8603

## Abstract

To achieve DNA sequencing using a solid-state nanopore, it is necessary to reduce the electric noise current. The noise current can be decreased by reducing the capacitance (*C*) of the nanopore device. However, we found that an electric-charge difference (Δ*Q*) between the electrolyte in one chamber and the electrolyte in another chamber occurred. For low capacitance devices, this electric-charge imbalance can lead to unexpectedly high voltage (Δ*V* = Δ*Q/C*) which disrupted the membrane when the two electrolytes were independently poured into the chambers. We elucidated the mechanism for the generation of initial defects and established new procedures for preventing the generation of defects by connecting an electric bypass between the chambers.

Nanopore DNA sequencing has attracted attention as a fourth-generation DNA sequencing method with potential advantages: long-read, high-throughput, and low-cost sequencing^1^. A device for nanopore DNA sequencing has a thin membrane with a nanopore separating two chambers filled with electrolyte. When a bias is applied across the membrane, an ionic current flows through the nanopore, and a DNA strand electrophoretically translocates through the nanopore. At this time, the ionic current is partially blocked. The blockade current differs in accordance with a convolution of four types of nucleotides (adenine, cytosine, thymine, and guanine), and the series of changes of the current determine the DNA sequence.

Based on their constituent materials, there are two types of nanopore technologies: biological^2^,^3^ and solid-state^4^,^5^,^6^,^7^,^8^,^9^,^10^,^11^,^12^,^13^,^14^,^15^,^16^,^17^,^18^,^19^,^20^,^21^,^22^,^23^,^24^,^25^,^26^,^27^,^28^,^29^. In biological nanopore technology, the four nucleotides are distinguished using the nanopores, such as mycobacterium smegmatis porin A^2^ and α-hemolysin (α-HL)^3^. Solid-state nanopore technology is based on inorganic materials, such as SiN, and thus, could potentially be robust and be applied in large-scale integration^27^. Venta *et al.* distinguished nucleotide homopolymers using a solid-state nanopore^17^. A long-term target of nanopore technology is to distinguish nucleotides at single-nucleotide resolutions.

One of the issues with solid-state nanopores in distinguishing nucleotides involves the reduction of ionic current noise^16^,^17^,^18^,^19^,^20^,^21^,^22^,^23^,^24^,^25^,^26^,^27^,^28^,^29^. The translocation velocity of DNA is high in the solid-state nanopore^20^, and a high sampling frequency is required to achieve single-nucleotide resolution^19^. By sampling the ionic current at high bandwidths, a high level of dielectric noise is generated^18^. In previous studies, the dielectric noise was reduced by decreasing the capacitance of the solid-state-nanopore devices^16^,^17^,^18^,^19^,^20^,^21^,^24^.

Recently, a novel process of nanopore fabrication was established using a dielectric breakdown induced by an applied high voltage^25^,^26^. This procedure can create nanopores without requiring a transmission electron microscope. We also established multilevel pulse voltage injection (MPVI) procedure to stably fabricate nanopores with diameters of 1 to 2 nm via dielectric breakdown^27^. These procedures provide new opportunities for nanopore researches.

In this study, we fabricated a low capacitance device for these procedures by coating dielectric material to the same devices used in^27^. The ionic current noise *I*_rms_ is largely dependent on the bandwidth, and the bandwidth must be high (>10 kHz) because the DNA translocation speed is less than 100 μs. In this high frequency regime, dominant noise is generated from the amplifier’s voltage noise (*V*_rms_)^22^, and the noise current is determined by the *V*_rms_ and the total impedance *Z*_all_ of the device. Figure 1(a) shows an equivalent circuit of a low capacitance device. *I*_rms_ can be calculated as follows:


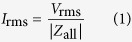



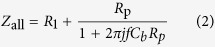


where *R*_l_ = 5 kΩ is the solution resistance, *R*_p_ = 100 MΩ is the nanopore resistance, *C*_b_ is the device capacitance, and *f* = 1 MHz is the bandwidth. According to Equation (1), *I*_rms_ can be reduced when |*Z*_all_| increases. We measured the capacitance and ionic current noise at a bandwidth of 1 MHz and confirmed that the noise decreased as the capacitance decreased. The correlation between noise current and capacitance is shown in Fig. 1(b). The black dots show the measured values, and the red line shows the theoretical fitted values assuming *V*_rms_ = 6 μV. As shown in Fig. 1(b), the capacitance should be maintained below 100 pF to sufficiently reduce the noise.

However, we also found that the thin membrane of the fabricated low capacitance device ruptured before the nanopores were fabricated. The membrane presented with initial defects after a potassium-chloride (KCl) electrolyte was poured onto both sides of the membrane. These initial defects have not been reported in the literature, and it is unknown why the defects occurred and how they could be prevented. In this study, we proposed a mechanism for the generation of these initial defects in the low capacitance devices. Furthermore, we devised new procedures for preventing the generation of the defects.

## Results and Discussion

### Investigation of the initial defects

There were the following possible causes of the initial defects: electrical breakdown or mechanical destruction such as the pressure of electrolytes flow and some stress in fabrication. To determine the causes of the initial defects, we investigated when the defects were generated by observing the membrane at each step of the fabrication procedure. Figure 2(a–d) show the procedure, which consists of four steps. First, dielectric material was coated near the membrane (Fig. 2(a)). The optical image of the fabricated device is shown in Figure S1. Second, flow cells were assembled with the device (Fig. 2(b)). Third, 90 μL of 1 M KCl electrolyte was poured into one chamber (Fig. 2(c)). Fourth, 90 μL of 1 M KCl electrolyte was poured into the other chamber (Fig. 2(d)). Figure 2(e–g) show the TEM images of the membranes as follows: (e), after the electrolyte was poured into one chamber; (f), after the electrolyte was poured into both chambers; and (g), an enlarged view of a defect. Ones of the defects are marked by red circles. These observations showed that the membrane did not have defects after the electrolyte was poured into one chamber. The defects were observed after the electrolyte was poured into both chambers. If the membrane had the defects in the fabrication process of the devices, the membrane should have been broken before the electrolyte was poured. Figure 2(g) shows that the defects were round pinhole-shaped with diameters ranging 1–10 nm. The defects didn’t have crack shape, and it also shows that the defects were generated not due to the pressure of electrolytes flow and the stress when dielectric materials were cured. These defects were similar to pores those were fabricated by applying a voltage to a membrane.

Next, we studied the correlation between the defects and device capacitance. We prepared 31 devices containing various amounts of dielectric material when we changed the coated area. The capacitance of each device was dependent on the volume of the dielectric material, and the capacitances differed with each other. The devices were put together into the flow cells, electrolytes were poured into the flow cells, and then the initial defects were generated. At last, we immersed two Ag/AgCl electrodes into the electrolytes in the two chambers, and measured the leakage current through the defects by applying 0.1 V (Fig. 3(a)). Figure 3(b) shows the correlation between the current at 0.1 V and the capacitance. The capacitance of each device was calculated from the measured current noise using Equations (1) and (2) assuming *V*_rms_ = 6 μV. By measuring and comparing the current at 0.1 V, we determined whether each device had defects or not.

The current *I*_a_ through a nanopore is calculated from the equation as follows^28^,^29^,^30^:


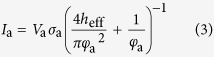


where *V*_a_ = 0.1 V is the applied voltage, *σ*_a_ = 0.105 S/cm is the conductance of the KCl at 22.5 °C, *h*_eff_ is the effective thickness of the nanopore, and *φ*_a_ is the diameter of the nanopore. *h*_eff_ was calculated to be 3.7 nm from our previous research^27^. From this Equation (3), when the current was 10 pA, the diameter of the nanopore was calculated to be 0.2 nm. This diameter of 0.2 nm is as small as one atom, and we defined the current of 10 pA as a threshold to determine whether the membrane had defects or not. In other words, when the current was greater than 10 pA, defects had been already generated before we started to measure the current. A large leakage current (>10 pA) flowed through the defects when the voltage of 0.1 V was applied. When the current was less than 10 pA, the membrane had no defects. The measured small current (<10 pA) was not attributed to a leakage current but was due to a noise current which mainly consists of dielectric noise^22^. As shown in Fig. 3(b), when the capacitance was 10–100 pF, the correlation coefficient between the leakage current and the capacitance was -0.7. As the capacitance decreased, more defects were generated and more leakage current flowed through the defects. This result shows that the defects were dependent on the impedance of the device, and the defects were due to the electrical breakdown.

### Mechanism of the initial defects generation

We proposed a mechanism for the generation of the initial defects. Figure 4(a–c) show the process by which the membrane ruptured. Figure 4(a) shows a schematic image after the electrolyte was poured into both chambers. An electric-charge difference was initially generated on surface charge and friction charge on the setups, and this charge was thought to be flowed into the electrolyte. Then, an electric-charge difference (Δ*Q* = |Δ*Q*_1_ −Δ*Q*_2_|) was generated between the electrolytes in the two chambers. The electric-charge difference generated a voltage (*V*) at the membrane (Fig. 4(b)). The generated voltage caused a dielectric breakdown and produced defects in the membrane (Fig. 4(c)). The voltage was calculated from the following equation:


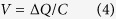


where *C* is the capacitance of the device. According to the equation, *V* increases as *C* reduces. Thus, as *C* was reduced, the resulting high voltage *V* generated more defects as shown in Fig. 2(f,g).

To verify this mechanism, we confirmed the presence of the electric charge difference and roughly estimated the electric-charge difference and the potential difference between two electrolytes. Figure 5(a,b) show the setup for measuring Δ*Q*. In this experiment, the electrolytes in both chambers were divided by a high capacitance device (*C* = 1000 pF) to not be broken by the electric-charge difference (Fig. 5(a)). First, we applied no voltage to the two electrodes and started to measure the current between two electrodes. Second, two electrodes were inserted into the chambers, and a current was flowed through the electrodes (Fig. 5(b)). Even though we applied no voltage to the membrane, we observed flowed current. This result shows that there were electric-charge imbalance and the imbalance generates the flowed current through the electrodes. Then, the current decreased due to the decreased electric-charge difference between the electrodes caused by a charge neutralization. The black line shows the transient current after the electrodes were inserted (Fig. 5(c)). The red line is an integral of the transient current after the electrodes were inserted. With this measurement in Fig. 5(c), the current was 0 pA and the value of the integral was stable after 60 s because the electric charge was neutralized. The integral at 60 s was thought to be the totality of the electric charge difference, and the calculated 80 pC was equivalent to Δ*Q*. We measured this electric charge difference four times, and the electric charge difference was calculated to be 120 ± 50 pC. The distribution was large because the electric charge was due to the occasional surface charge and friction charge. It is thought to be why the plots of initial leakage current were scattered in Fig. 3(b).

We estimated the voltage *V* at the membrane from an 120 pC electric-charge difference Δ*Q*, which is dependent on the capacitance *C* of the device (Equation (1)). We assumed that the capacitances of the nanopore devices with and without dielectric films were 10 pF and 1000 pF, respectively.

When a low capacitance device is operating (i.e., *C* = 10 pF), *V* is 12 V. The calculated voltage *V* of 12 V generates high electric field (1.2 V/nm) across the 10-nm-thick SiN membrane. The order of ~1 V/nm is high electric field to disrupt a SiN membrane^25^, and 90% of nanopores were generated within 10 s when 0.7 V/nm was applied to our 10-nm-thick SiN membrane^27^. In our measurement procedure, we poured electrolytes into flow cells, and in 1–5 min we inserted two electrodes to the flow cells and started to measure the initial leakage current. Once we started the measurement, the electric charge difference was decreased through the electrodes and new defects were not generated. The period of 1–5 min was also sufficient to break the membrane. On the other hand, when a high capacitance device is operating (i.e., *C* = 1000 pF), the electric field is calculated to be 0.012 V/nm. The order of ~0.01 V/nm is too low to disrupt a SiN membrane. When we applied ~0.1 V/nm to three high capacitance devices for 10 min, all the membranes were not broken. It is why the high capacitance devices have no defects.

### Prevention of the generation of initial defects

To prevent the generation of the initial defects, we developed a new charge neutralization procedure. In the conventional procedure described in Fig. 2(a–d), the electric-charge difference cannot be decreased because the two electrolytes are poured separately, which generates initial defects in the membrane. In the new procedure, the electric-charge difference can be decreased using bypass wiring. Figure 6 shows the setup procedure. First, the bypass wiring was connected between the chambers, and electrolyte was poured into one chamber (Fig. 6(a)). Second, electrolyte was poured into the other chamber (Fig. 6(b)). As the electrolyte was being poured into the other chamber, the electrolyte solutions in both chambers were connected via the bypass wiring and the electric-charge difference was decreased. Third, both chambers were filled with electrolyte (Fig. 6(c)). In this manner, the bypass wiring decreased the electric-charge difference before the electrolyte had contacted the other side of the membrane. Finally, a measuring circuit was connected between the electrolytes, thereby generating an electric-charge difference, and the bypass wiring was removed (Fig. 6(d)). A bypass channel can be substituted for the bypass wiring to reduce the electric-charge difference. This other procedure is explained in “Supplementary Section SI-2”.

After the charge neutralization was completed, we confirmed that the charge-neutralized device had no defects using the same experiment as previously explained in Fig. 3(a). Figure 7 shows the correlation between the current and capacitance using non-charge-neutralized and charge-neutralized devices. The black dots indicate the non-charge-neutralized devices and the red dots indicate the charge-neutralized devices. The current measured with the charge-neutralized devices was negligible (less than 10 pA) and thus, was not attributed to a leakage current. Figure 7 shows that the charge-neutralized devices have no defects in the membrane, even when the capacitance was reduced (10–100 pF). We thus conclude that the charge neutralization prevented the generation of the initial defects.

### ssDNA translocation using charge-neutralized low capacitance devices

We measured an ionic-current blockade during single-stranded DNA (ssDNA) translocation using the charge-neutralized low capacitance device and confirmed that the device can be used for measuring an ionic-current blockade with low noise. First, we fabricated a low capacitance device with a capacitance of 20 pF and set up an experiment with charge neutralization procedure. Then, a nanopore with a diameter of 2 nm was fabricated using MPVI with sub-nanometer precision^27^. The electrolyte in the *cis* chamber was replaced with 1 M KCl electrolyte with a 60-mer, single-stranded poly(dA). Figure 8 shows the ionic-current through the nanopore at 0.3 V, and ssDNA-translocation events were detected. When the data were filtered at 100 kHz, the ionic current included high noise values of 300 pA_rms_ when using a high capacitance device with a capacitance of 1000 pF (Fig. 8(a)). The noise was reduced to 30 pA_rms_ by using a charge-neutralized low capacitance device with a capacitance of 20 pF (Fig. 8(b)).

## Conclusion

A new charge neutralization procedure was developed to prevent the generation of initial defects by the electric-charge difference. The electric-charge difference Δ*Q* = 120 pC occurred when electrolytes were separately poured into the two chambers of a nanopore device. The resultant electric-charge difference was sufficiently large to break the membrane. To decrease the electric-charge difference, we developed the charge neutralization procedure to prevent the generation of the initial defects. When using conventional procedures without charge neutralization, 20 of 27 fabricated low capacitance devices (*C* < 100 pF) were broken. On the other hand, when charge neutralization was employed, none of the eight fabricated low capacitance devices (*C* < 100 pF) were broken.

The charge neutralization can decrease the electric-charge differences generated during electrolyte pouring and when other procedures are performed, e.g., connecting electrodes and displacing electrolytes. We observed that these procedures also generated initial defects or widened the diameters of the nanopores.

Some previous studies showed that the dielectric noise could be reduced by decreasing the capacitance, and there were no studies reporting any initial defects. This observed initial defect was due to the capacitance, the thickness, and the electric-charge difference between two electrolytes. The electric-charge difference can be different on each laboratory, and the ultra-thin membranes of the ultra-low capacitance devices are in a relatively new area of the exploration. We speculate that it is why there were no reports about these initial defects.

The origin of the electric-charge difference was not sure. We used electrostatic protection procedure with anti-static gloves and stands, but the initial defects could not be prevented. We thus consider that the electric-charge difference was due to initial surface charge of the flow cells and friction charge when we put together the setups.

## Methods

### Fabrication of low capacitance devices

The low capacitance devices were fabricated by coating dielectric film layers adjacent the thin SiN membranes. The 10-nm-thick membranes (approximately 500 × 500 nm^2^ square) were supported on 725-μm-thick Si substrates. On the front surfaces of the membranes, SiN/SiO_2_ (100/300 nm) layers were deposited to prevent physical disruptions to the thin membranes.

The substrates adjacent the thin membranes were coated with dielectric films, such as silicone elastomer and polyimide with thicknesses of 5–10 μm, by spreading out a drop of uncured silicone elastomer and polyimide under a stereoscopic microscope. The coated area and the thickness were determined with confocal laser scanning microscope.

The coated silicone elastomer and polyimide were cured in thermostat chambers for 30 min in 110 °C and for 30 min in 300 °C, respectively. Before an ionic current was measured, the device was hydrophilized and cleaned on each side with argon/oxygen plasma (SAMCO, Inc., Kyoto, Japan) at a flow rate of 20 sccm, a pressure of 20 Pa for 45 s, and a power of 10 W.

### Observations of initial defects by TEM

The initial defects were observed using a JEM-2100F (HRP) TEM (JEOL, Ltd., Tokyo, Japan) at an accelerating voltage of 200 keV. Before the observations, the membranes were washed with pure water to remove any residual salts from the electrolytes. First, we took the devices from flow cells, washed the devices and dipped the devices in pure water for 10 min at least. Then, we took the devices and blew off water drop adhered to the devices.

### Measurement of the leakage current and capacitance

**Figure 1:**
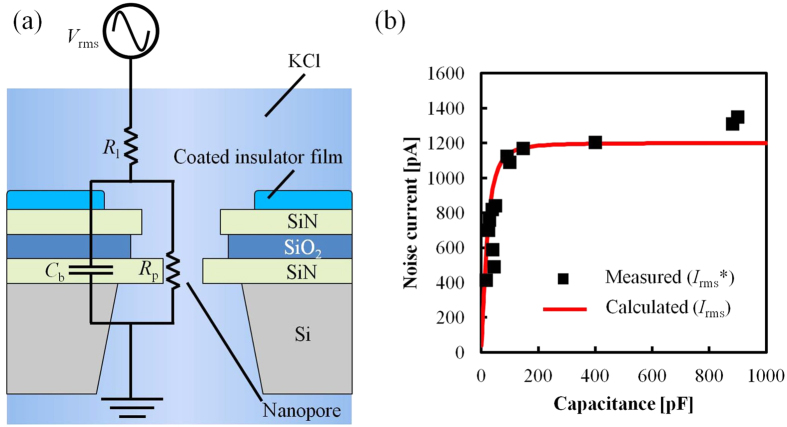
Measurement of the noise current. (**a**) Representative circuit of the measurement system for calculating the noise current *I*_rms_. (**b**) Noise current versus capacitance. Black dots are the measured values, and the red line is the theoretical values assuming *V*_rms_ = 6 μV.

**Figure 2:**
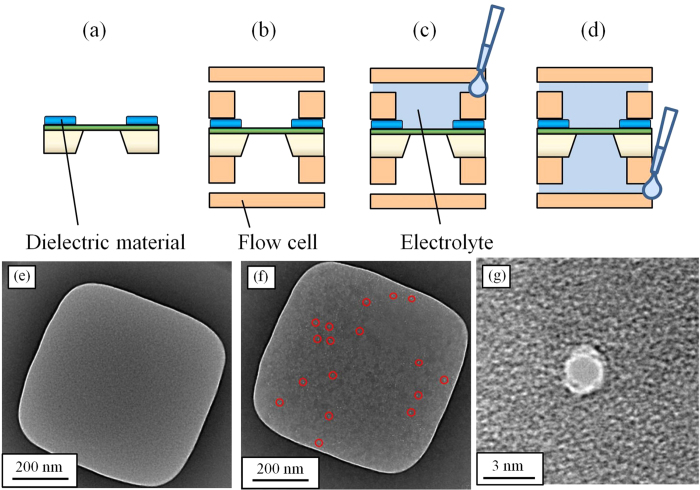
TEM images of the membranes at each setup step. (**a**–**d**) show the setup procedure, and (**e**–**g**) show the TEM images of the membranes. (**a**) Coating the insulator film on the substrate. (**b**) Combining the setup with the device and flow cells. (**c**) Pouring KCl electrolyte into a chamber of the flow cell. (**d**) Pouring KCl electrolyte into the other chamber. (**e**) Image of a device after KCl electrolyte is poured into one chamber. (**f**) and (**g**) Images after KCl electrolyte is poured into both chambers. Ones of the defects are marked by red circles in (**f**). (**g**) is an enlarged view of one of the defects which is shaped like a pinhole structure.

**Figure 3:**
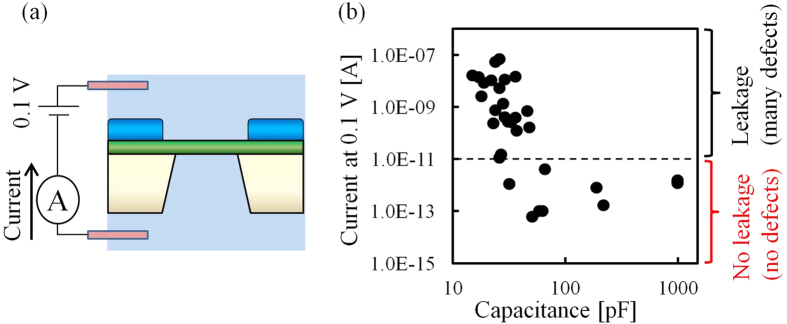
Correlation between the leakage current and capacitance. (**a**) Setup for the measurement of the leakage current. (**b**) Initial leakage current (|*I*_ini_*|) (log scale) at 0.1 V versus capacitance. When the current is more than 10 pA, defects are generated on the membrane, and a large leakage current flows through the defects. When the current is less than 10 pA, no defects are generated on the membrane, and the measured small current is noise current.

**Figure 4:**
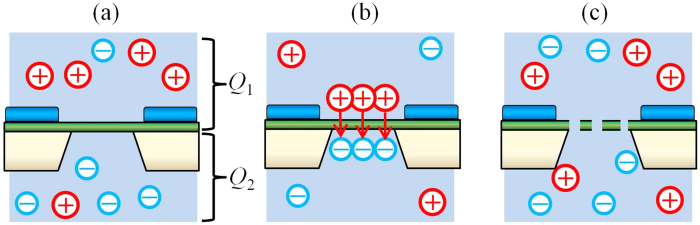
Schematic images of hypothetical mechanism for the generation of initial defects. (**a**) An electric-charge difference exists between the electrolytes in the chambers. (**b**) The electric-charge difference produces high voltage at the membrane. (**c**) The high voltage causes dielectric breakdown, and the dielectric breakdown creates defects in the membrane.

**Figure 5:**
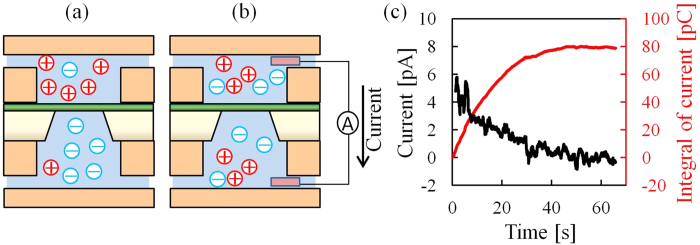
Measurement of the electric-charge difference. (**a**,**b**) show schematics of the setup for the measurement of the electric-charge difference. (**a**) There is an electric-charge difference between the electrolytes in the chambers. Two electrolytes are divided by a high capacitance device. (**b**) Two electrodes are inserted into the electrolytes, and current flows through the electrodes. (**c**) Plot of a transient current through the electrodes as a function of time. The black line shows the transient current, and the red line shows the integral of the current.

**Figure 6:**
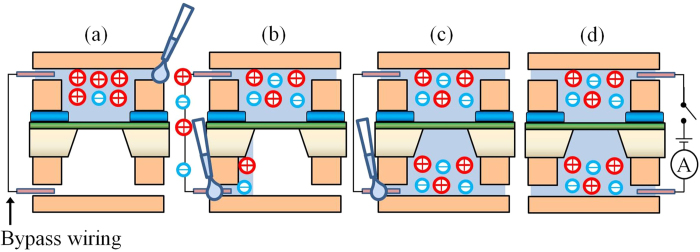
Setup procedure with charge neutralization. (**a**) Bypass wiring was connected between the chambers, and the electrolyte was poured into one chamber. (**b**) The electrolyte was poured into the other chamber. As the electrolyte was being poured into the chamber, the electrolyte was connected to the wiring. (**c**) The chambers were filled with electrolytes. (**d**) After a measuring circuit was connected to the electrolytes, the bypass wiring was removed.

**Figure 7:**
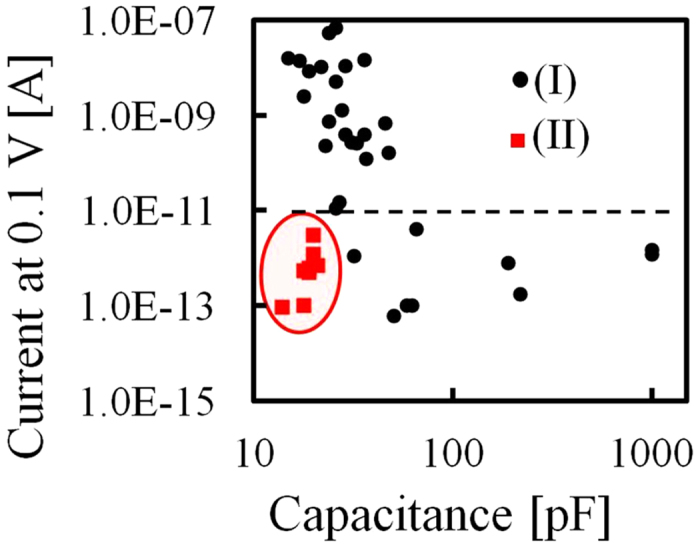
Correlation between the leakage current and the capacitance of devices with and without charge neutralization. The graph shows the initial leakage current (|*I*_ini_*|) (log scale) at 0.1 V versus capacitance of (I) non-charge-neutralized devices and (II) charge-neutralized devices. Unlike the non-charge-neutralized devices, the charge-neutralized devices had no leakage current (<0 pA) in the membrane, even when the capacitance was less than 100 pF.

**Figure 8:**
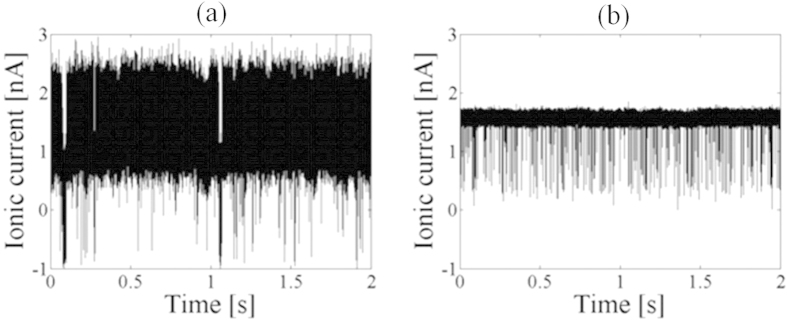
Measurement of ssDNA translocation events through the nanopores. The translocation events of 60-mer, single-stranded poly(dA) through a nanopore with a diameter of 2 nm at 300 mV. (**a**,**b**) Time traces of a non-charge-neutralized, high capacitance device (1000 pF) and a charge-neutralized, low capacitance device (20 pF), respectively. The data were low-pass filtered at 100 kHz.

To evaluate the correlation between the leakage current and the capacitance of the devices (in Figs 3(b) and 7), the leakage current was measured using a 4156B precision semiconductor parameter analyzer (Agilent Technologies, Inc., Santa Clara, CA, USA). The capacitances were calculated using Equations (1) and (2) and data from the noise current measured by a VC100 low-noise voltage-clamp amplifier (Chimera Instruments, LLC, New York, NY, USA). Figure 1(b) shows the capacitances as measured by a 4294A precision impedance analyzer (Agilent Technologies, Inc., Santa Clara, CA, USA).

### Fabrication of the nanopores and measurement of the blockade current

To fabricate the nanopores, MPVI was applied with a 41501B SMU and Pulse Generator Expander (Agilent Technologies, Inc., Santa Clara, CA) and the 4156B precision semiconductor parameter analyzer. The blockade current of the translocation of the ssDNA was measured with the VC100 low-noise voltage clamp amplifier.

## Additional Information

**How to cite this article**: Matsui, K. *et al.* Prevention of Dielectric Breakdown of Nanopore Membranes by Charge Neutralization. *Sci. Rep.* **5**, 17819; doi: 10.1038/srep17819 (2015).

## Supplementary Material

Supporting Information
