# Supplementary material for: Prevention of Dielectric Breakdown of Nanopore Membranes by Charge Neutralization
Source: Sci Rep. 2015 Dec 4;5:17819. doi: 10.1038/srep17819 (PMC4669463; doi:10.1038/srep17819)
Supplement: Supporting Information [file srep17819-s1.doc]

**Supplementary Information**

**Prevention of Dielectric Breakdown of Nanopore Membranes by Charge Neutralization**

Kazuma Matsui*^1^, Itaru Yanagi^1^, Yusuke Goto^1^, and Ken-ichi Takeda^1^

^1^Hitachi, Ltd., Research & Development Group, Center for Technology Innovation - Healthcare,

1-280 Higashi-Koigakubo, Kokubunji, Tokyo, 185-8603

**SI-1. Design of the low capacitance device**

The low capacitance device was fabricated by coating silicone elastomer and polyimide near the membrane. Figure S1 shows images of the fabricated device when we used silicone elastomer as the dielectric material. Figure S1(a) is a top optical view of the nanopore device. Figure S1(b) is a schematic of a cross-section of the device.

**Figure S1 | Fabricated low capacitance devices.** (a) A top optical view of a low capacitance device. (b) A schematic of a cross-section of the low capacitance device. A 5 – 10 μm-thick silicone elastomer layer, a 100 nm-thick SiN layer, a 300 nm-thick SiO_2_ layer, and a 10 nm-thick SiN layer were deposited on a 725 μm-thick Si substrate.

**SI-2. Charge neutralization using a bypass channel**

An alternative charge neutralization procedure using bypass channel can also prevent the generation of initial defects. Figure S2 shows the setup. First, a bypass channel was connected between the chambers (Figure S2(a)). Second, both chambers were filled with the same electrolyte via the bypass channel (Figure S2(b)). Before the electrolyte contacted both sides of the membrane, the electric-charge difference had already been decreased. Third, two electrodes for measuring the ionic current were connected to the electrolyte in both chambers. Finally, the bypass channel was removed (Figure S2(c)). This procedure also can neutralize the electrolytes in both chambers.

**Figure S2 | Setup procedure using bypass channel.** (a) A bypass channel was connected between the chambers, and the electrolyte was poured into one chamber. (b) The electrolyte filled the other chamber through the bypass channel. (c) After a measuring circuit was connected to the electrolytes, the bypass channel was removed.
